# Cardiac Arrest Secondary to Hypercapnia in an Obese Patient: A Case of Unresponsiveness With Preserved Awareness

**DOI:** 10.7759/cureus.98800

**Published:** 2025-12-09

**Authors:** Kajal S Shah, Ibtesam A Bhatti, Hinzilah S Minhas, Sunho Judy Lee, Hassan Jafary

**Affiliations:** 1 Internal Medicine, Avalon University School of Medicine, Willemstad, CUW; 2 Medicine, Stanaford Medical Clinic, Beckley, USA

**Keywords:** acute non-st-elevation myocardial infarction, acute respiratory failure, cardiac arrest, hypercapnia, obesity hypoventilation syndrome, obstructive sleep apnea, pneumonia, syncope, unresponsiveness, ventricular fibrillation

## Abstract

In rare circumstances, hypercapnia can precipitate life-threatening cardiac events, particularly in patients with obesity hypoventilation syndrome and obstructive sleep apnea (OSA). Awareness during such episodes of unresponsiveness is uncommon, underscoring the complex interaction between respiratory and neurologic physiology. A 58-year-old Caucasian female with a body mass index of 37.4 kg/m², OSA, and a history of recurrent syncope presented to the emergency room with chest pain and shortness of breath. During hospitalization, she received sublingual nitroglycerin, after which she developed bradycardia and was administered atropine. She subsequently progressed into ventricular tachycardia and required two defibrillations to restore sinus rhythm. Remarkably, during resuscitation, she reported awareness despite unresponsiveness, hearing staff, and feeling defibrillation shocks. A comprehensive cardiac evaluation, including catheterization, troponin trends, ECG, imaging, echocardiography, Doppler studies, cultures, and laboratory tests, revealed no acute coronary pathology. She was admitted to the intensive care unit, underwent implantable cardioverter-defibrillator (ICD) placement, and was later discharged. Several days after discharge, she experienced another syncopal episode at home, this time with complete unresponsiveness and no awareness. On readmission, arterial blood gas analysis revealed significant hypercapnia, prompting initiation of bilevel positive airway pressure therapy. She improved clinically and was discharged on continuous positive airway pressure therapy for OSA, along with weight management counseling. This case emphasizes the importance of recognizing hypercapnia-induced cardiac events in obese patients with severe OSA. Identifying this mechanism, particularly when presenting as unresponsiveness with preserved awareness, highlights the diagnostic challenges and the necessity for vigilant monitoring, non-invasive ventilation, ICD support, and lifestyle modification to prevent recurrence and improve long-term outcomes.

## Introduction

Obesity hypoventilation syndrome (OHS) is defined by the triad of obesity (body mass index (BMI) ≥30 kg/m²), chronic daytime hypercapnia (PaCO₂ >45 mmHg), and sleep-disordered breathing, most commonly obstructive sleep apnea (OSA) [1,2]. Excess adiposity imposes mechanical loads on the chest wall and blunts central ventilatory drive, predisposing patients to chronic carbon dioxide retention and recurrent episodes of acute-on-chronic hypercapnic respiratory failure [3].

Although hypercapnia is typically viewed as a marker of respiratory insufficiency, emerging evidence suggests that elevated PaCO₂ can have profound cardiovascular effects, including increased pulmonary vascular resistance, right ventricular strain, autonomic dysregulation, and arrhythmogenesis [4]. In patients with OHS and OSA (conditions already associated with sympathetic activation, endothelial dysfunction, and sudden cardiac death), acute hypercapnic decompensation creates a vulnerable milieu in which even modest derangements in ventilation may precipitate ventricular tachyarrhythmias or cardiac arrest [2,5].

A less recognized but clinically important phenomenon is preserved awareness during apparent unresponsiveness, in which patients retain auditory or tactile perception despite exhibiting outward unresponsiveness. Similar states have been described in peri-arrest physiology, syncope with transient cerebral hypoperfusion, and certain anesthetic awareness events, suggesting that cerebral cortical function may persist even when motor responsiveness is lost [6]. This phenomenon is rarely reported in the context of hypercapnia-induced cardiac instability and may lead clinicians to underestimate a patient’s perceptual awareness during resuscitative interventions.

We present the case of an obese woman with severe OSA who experienced a hypercapnia-related cardiac arrest and later described detailed awareness during a period of clinical unresponsiveness. This report underscores the importance of recognizing hypercapnia as a potential trigger for malignant arrhythmias in high-risk patients and highlights the need to consider preserved awareness during resuscitation, a feature that remains underreported in the literature.

## Case presentation

A 58-year-old Caucasian woman presented to the emergency department complaining of chest pain and shortness of breath. Her medical history was significant for morbid obesity (BMI: 37.4 kg/m²), hyperlipidemia, cardiac muscle bridging, OSA, chronic respiratory failure, prior pulmonary embolism, coronary artery disease, and recurrent syncope. She described her chest pain as radiating to the left neck and left arm. At baseline, she required continuous supplemental oxygen at home as prescribed by her primary care physician.

Upon arrival, her vital signs were stable: blood pressure, 138/75 mmHg; pulse, 92 beats/minute; respiratory rate, 19 breaths/minute; and oxygen saturation, 97% on a nasal cannula. She rated the pain as 6/10 at the time of evaluation, reduced from 8/10 at presentation. Physical examination revealed no acute distress, and cardiac auscultation was unremarkable.

**Figure 1:**
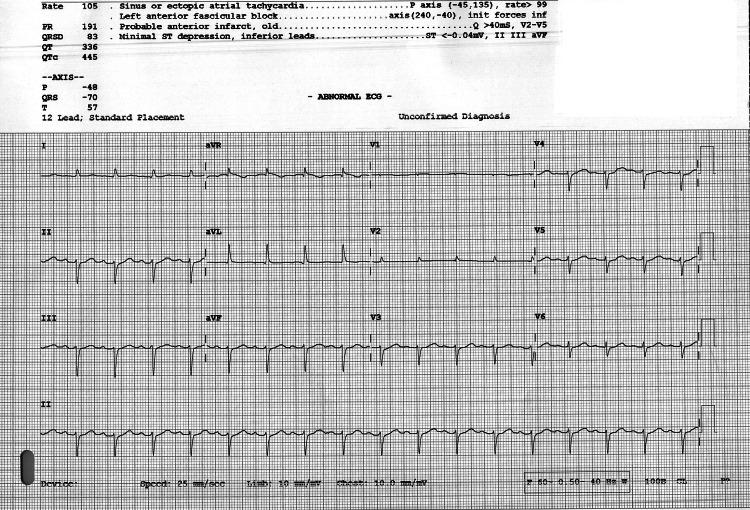
Twelve-lead ECG showing sinus or ectopic atrial tachycardia with Q waves in leads V2–V5 and minimal ST depression in the inferior leads. Twelve-lead ECG demonstrating sinus or ectopic atrial tachycardia at approximately 105 beats/minute, Q waves in leads V2–V5 consistent with an old anterior myocardial infarction, and minimal ST-segment depression in the inferior leads (II, III, aVF). Left anterior fascicular block is also noted. These findings suggested a prior anterior infarction but no evidence of acute ischemia contributing to the arrest. ECG = electrocardiogram; PR = PR interval; QRSD = QRS duration; QT = QT interval; QTC = corrected QT interval; P = P wave; QRST = ventricular depolarization complex; T = T wave; P-axis = atrial electrical axis; QRS-axis; ventricular electrical axis; T-axis = ventricular repolarization axis

A 12-lead ECG (Figure 1) showed sinus or ectopic atrial tachycardia at 105 beats/minute, Q-waves in leads V2-V5 consistent with an old anterior infarct, and minimal ST-segment depression in the inferior leads (II, III, aVF). Relevant laboratory results are summarized in Table 1.

**Table 1:** Routine laboratory investigations on presentation. Routine laboratory investigations on admission show normal complete blood count and electrolyte levels. Renal function is preserved (eGFR >90 mL/minute/1.73 m²), and cardiac biomarkers are within normal limits. Mild hyperglycemia is noted (glucose: 215 mg/dL). Serial troponin assays are negative, ruling out acute myocardial injury. WBC = white blood cell; BUN = blood urea nitrogen; GFR = glomerular filtration rate; BNP = B-type natriuretic peptide; LVEF = left ventricular ejection fraction

Laboratory test	Result	Reference range	Units
WBC	7.51	4.0–9.7	×10^3^/µL
Hemoglobin	13.2	12.0–16.0	g/dL
Platelet count	240	182–369	×10^3^/µL
Sodium	139	136–145	mmol/L
Potassium	4.6	3.5–5.1	mmol/L
BUN	12	9–23	mg/dL
Creatinine	0.76	0.55–1.02	mg/dL
GFR	>90	>90	mL/minute/1.73m^3^
Glucose	215	74–106	mg/dL
BNP	83	0–125	pg/mL
Troponin	Negative × 3	Negative	pg/mL
LVEF	60%	55–70	%

During hospitalization, the patient developed recurrent chest pain without ST elevation but with non-specific ST-T abnormalities. She subsequently became bradycardic, received epinephrine, progressed to ventricular tachycardia, and required two external defibrillation shocks. Throughout these events, she remained alert and oriented. She was transferred to the intensive care unit (ICU) awake, with an oxygen saturation of 80-82% on a non-rebreather mask. Bilevel positive airway pressure (BiPAP) was initiated. An intravenous heparin infusion was started, and 80 mg of IV furosemide was administered for volume management. Arterial blood gas (ABG) analysis was performed (Table 2). She was scheduled for left-heart catheterization.

**Table 2:** Serial arterial blood gas analyses showing progressive improvement in respiratory acidosis over hospitalization. Results represent serial arterial samples collected at different time points during hospitalization. The first two samples were obtained several minutes apart on the same day, the third on the following day, and the final sample was obtained approximately nine days later upon readmission. Exact collection dates have been omitted to maintain patient confidentiality. Reference ranges are provided in the last column. Arrows indicate deviation from normal values (↑ = above reference range; ↓ = below reference range). Findings are consistent with compensated respiratory acidosis and improving gas exchange. ABG = arterial blood gas; PaCO₂ = partial pressure of carbon dioxide; PaO₂ = partial pressure of oxygen; HCO₃⁻ = bicarbonate; CO₂ = carbon dioxide; O₂ = oxygen; BiPAP = bilevel positive airway pressure

Parameter	Initial (T_0_)	Repeat (T_1_)	Next day (T_2_)	Nine days later (T_3_)	Reference range	Units
pH	7.32 ↓	7.3 ↓	7.38	7.4	7.35–7.45	-
PaCO_2_	60 ↑	58 ↑	56 ↑	53 ↑	35–45	mmHg
PaO_2_	94	73 ↓	107 ↑	96	80–100	mmHg
HCO_3_^-^	30.9 ↑	28.5 ↑	33.1 ↑	32.8 ↑	22–26	mEq/L
Total CO_2 _	32.7 ↑	30.30 ↑	34.80 ↑	34.40 ↑	23–30	mEq/L
O_2_ saturation	97	93 ↓	98 ↑	98	95–100	%
Base excess	+3 ↑	–	+6 ↑	+7 ↑	-2 to +2	mEq/L)
BiPAP	5	10	10	-	-	cmH_2_O

**Figure 2:**
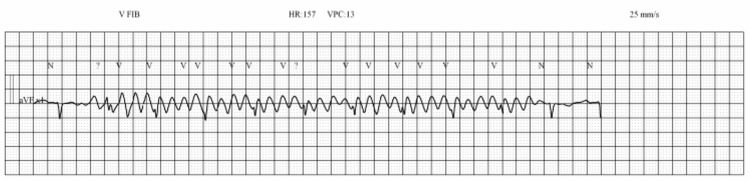
Ventricular fibrillation requiring defibrillation. Cardiac rhythm strip demonstrating ventricular fibrillation characterized by rapid, irregular, and chaotic ventricular waveforms with no discernible P waves, QRS complexes, or T waves. The tracing reflects pulseless ventricular fibrillation at an approximate rate of 150–160 beats per minute. Immediate defibrillation was performed, and the patient subsequently regained sinus rhythm and hemodynamic stability. This rhythm explained the patient’s sudden collapse and the need for emergent defibrillation. ECG = electrocardiogram; HR = heart rate; V FIB = ventricular fibrillation; VPC = ventricular premature complex; BPM = beats per minute

Later during the admission, she experienced severe recurrent chest pain, received sublingual nitroglycerin, and a code-blue event was activated. Cardiopulmonary resuscitation (CPR) was initiated, and atropine was administered for bradycardia. The rhythm evolved into ventricular tachycardia with a pulse (Figure 2), requiring two rounds of defibrillation. She subsequently regained sinus tachycardia with systolic blood pressures in the 160s and remained responsive. She was transferred back to the ICU without requiring intubation. Given the recurrence of ventricular arrhythmias, she was fitted with a LifeVest wearable cardioverter-defibrillator and referred to electrophysiology for evaluation for an implantable cardioverter-defibrillator.

**Figure 3:**
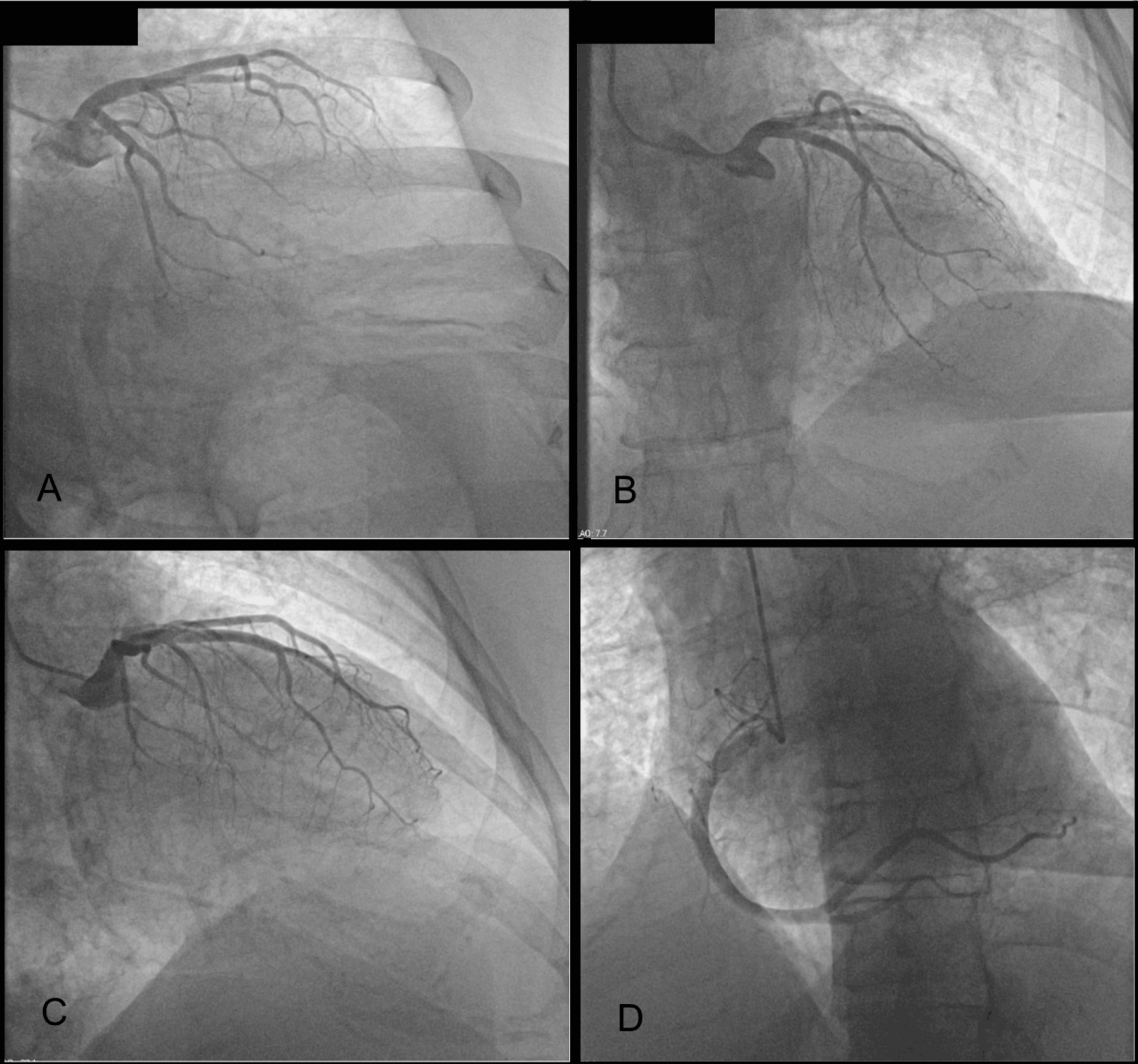
Coronary angiography demonstrating non-obstructive coronary artery disease. (A–D) Sequential coronary angiographic projections showing normal coronary flow without hemodynamically significant stenosis. (A) Circumflex artery showing preserved patency and small-caliber distal segment without discrete narrowing. (B–C) Left anterior descending artery demonstrating mild non-obstructive myocardial bridging and a small-caliber distal segment. (D) Right coronary artery showing a dominant vessel with smooth contour and less than 40% stenosis of the right posterior descending artery. Collectively, these findings are consistent with non-obstructive coronary artery disease involving the small vessels, as reported on catheterization.

**Figure 4:**
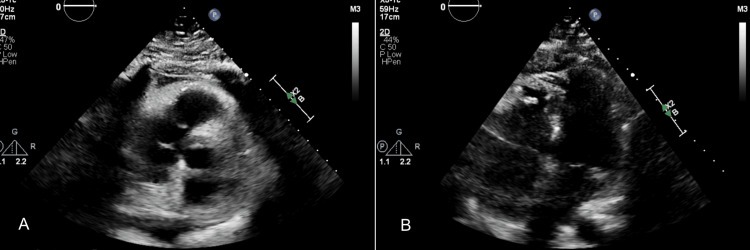
Transthoracic echocardiography demonstrating normal left ventricular function. (A–B) Standard transthoracic echocardiographic images showing preserved left ventricular systolic function. (A) Parasternal long-axis view demonstrating a normal-sized left ventricle with mild concentric hypertrophy, normal wall motion, and no regional wall motion abnormalities. (B) Apical four-chamber view showing normal chamber sizes, normal septal and lateral wall motion, and a left ventricular ejection fraction of ≈60%. A small pericardial effusion is present without evidence of tamponade. Valvular assessment shows mild aortic regurgitation and trace tricuspid regurgitation, with no hemodynamically significant stenosis. These findings confirm preserved global systolic function and support a diagnosis of small-vessel (microvascular) coronary disease rather than obstructive epicardial disease.

Comprehensive cardiac evaluation, including left-heart catheterization, serial troponins, ECG, echocardiography, and laboratory testing, revealed non-obstructive coronary artery disease with probable small-vessel involvement. Coronary angiography demonstrated normal coronary flow without hemodynamically significant stenosis (Figure 3). Transthoracic echocardiography showed preserved left ventricular systolic function and no regional wall motion abnormalities (Figure 4).

**Figure 5:**
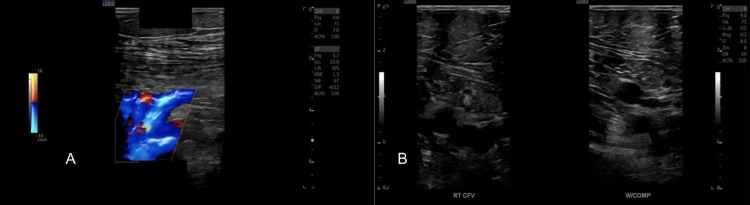
Lower etremity venous Doppler ultrasound demonstrating normal flow and compressibility. (A) Color Doppler ultrasound demonstrating normal venous flow and patent lumen without evidence of thrombus. (B) B-mode compression ultrasonography of the right common femoral vein before and during compression, showing complete vein collapse with applied pressure, confirming normal venous patency. CFV = common femoral vein; COMP = compression

To further evaluate alternative causes of respiratory compromise, bilateral lower extremity venous Doppler ultrasonography was performed, demonstrating normal venous compressibility and flow without evidence of deep vein thrombosis (Figure 5). The patient remained hemodynamically stable and continued BiPAP therapy under pulmonology supervision for hypercapnia management. Post-catheterization vital signs included blood pressure of 155/78 mmHg, pulse of 98 beats/minute, respiratory rate of 24 breaths/minute, and oxygen saturation of 93% on 4 L nasal cannula.

Neurology was consulted for intermittent slurred speech and chronic dysphagia. Prior swallow studies revealed no abnormalities requiring intervention or associated with an increased risk of aspiration. A 72-hour electroencephalograpy and CT head were negative for seizure activity or acute neurologic insult.

Several days after discharge, the patient experienced another syncopal episode at home and was found unresponsive with no awareness of her surroundings. On readmission, ABG analysis (Table 2) revealed significant hypercapnia, prompting initiation of BiPAP therapy, which led to clinical improvement. After stabilization, she was discharged on continuous positive airway pressure (CPAP) therapy for OSA, weight-management counseling, and follow-up with cardiology and pulmonology specialists.

## Discussion

Hypercapnia is defined as an elevation of the arterial partial pressure of carbon dioxide (PaCO₂) above 45 mmHg. Carbon dioxide (CO₂), a metabolic byproduct of cellular respiration, is normally eliminated through diffusion across the alveolar-capillary membrane. When this process is impaired, CO₂ accumulates, resulting in respiratory acidosis.

Obesity is a major contributor to chronic hypercapnia. Excess adiposity alters respiratory mechanics by increasing chest wall load, reducing lung and chest wall compliance, and elevating airway resistance, all of which increase the work of breathing. These changes contribute to OHS, wherein impaired ventilatory drive and mechanical restriction lead to chronic CO₂ retention. Leptin resistance, common in obesity, further blunts central respiratory drive, exacerbating hypoventilation and hypercapnia [7]. As CO₂ levels rise, the respiratory system attempts to compensate through hyperventilation; however, this compensation may be insufficient, leading to persistent respiratory acidosis [8].

Chronic hypercapnia has significant cardiovascular consequences. Elevated CO₂ causes pulmonary vasoconstriction, increased pulmonary artery pressures, and right ventricular hypertrophy, eventually progressing to right-sided heart failure [9]. Acidosis impairs myocardial contractility, contributing to hypotension and reduced cerebral perfusion, which may manifest as syncope. Hypercapnia also triggers sympathetic activation, increasing myocardial oxygen demand and predisposing to malignant ventricular arrhythmias, consistent with our patient’s progression from bradycardia to ventricular tachycardia [3]. In individuals with OHS or OSA, autonomic dysregulation, endothelial dysfunction, and systemic inflammation amplify cardiovascular risk [9].

CO₂ narcosis represents another severe manifestation of hypercapnia, driven by cerebral vasodilation in response to elevated PaCO₂. Clinically, this can range from confusion and lethargy to coma. In our patient, significant hypercapnia, together with OHS and OSA, likely impaired cerebral perfusion and contributed to her syncopal episode [10].

Awareness during cardiac arrest is rare but clinically recognized. Transient cerebral perfusion during chest compressions or brief periods of spontaneous circulation may preserve partial consciousness even when systemic circulation is severely compromised [11]. Patients may appear unresponsive yet retain perceptual awareness or recall of resuscitative efforts. In this case, the patient’s awareness during defibrillation is most plausibly explained by intermittent restoration of cerebral blood flow sufficient to maintain consciousness despite ongoing cardiac instability [12].

This case underscores the importance of early recognition and prompt correction of hypercapnia in obese patients who present with unexplained respiratory or neurologic symptoms. Non-invasive ventilatory support, such as BiPAP, plays a central role in improving alveolar ventilation and reducing PaCO₂ [1]. In our patient, rapid initiation of BiPAP stabilized gas exchange and prevented further cardiovascular deterioration. Preventive strategies should prioritize optimization of OSA therapy, structured weight reduction interventions, and careful oxygen titration to avoid suppressing hypoxic ventilatory drive.

## Conclusions

This case illustrates the complex interplay between obesity, hypoventilation, and cardiovascular instability. In individuals with OHS and OSA, acute CO₂ retention may precipitate hemodynamically significant ventricular tachyarrhythmias or cardiac arrest. The patient’s preserved awareness during apparent unresponsiveness highlights a rarely described phenomenon and expands the clinical understanding of perceptual experiences during peri-arrest states. Recognizing hypercapnia as a potential contributor to unexplained arrhythmias or syncope is essential, particularly in patients with severe obesity and sleep-disordered breathing. Early application of non-invasive ventilation, careful cardiac monitoring, and long-term weight management remain critical to preventing recurrence and improving patient outcomes.
